# NFIL3 mutations alter immune homeostasis and sensitise for arthritis pathology

**DOI:** 10.1136/annrheumdis-2018-213764

**Published:** 2018-12-14

**Authors:** Susan Schlenner, Emanuela Pasciuto, Vasiliki Lagou, Oliver Burton, Teresa Prezzemolo, Steffie Junius, Carlos P Roca, Cyril Seillet, Cynthia Louis, James Dooley, Kylie Luong, Erika Van Nieuwenhove, Ian P Wicks, Gabrielle Belz, Stéphanie Humblet-Baron, Carine Wouters, Adrian Liston

**Affiliations:** 1 Department of Microbiology and Immunology, KUL - University of Leuven, Leuven, Belgium; 2 VIB Center for Brain and Disease Research, Leuven, Belgium; 3 Walter and Eliza Hall Institute of Medical Research, Parkville, Victoria, Australia; 4 Department of Medical Biology, University of Melbourne, Parkville, Victoria, Australia; 5 Department of Pediatrics, University Hospitals Leuven, Leuven, Belgium

**Keywords:** NFIL3, juvenile idiopathic arthritis, IL-1β, macrophages, genetic

## Abstract

**Objectives:**

*NFIL3* is a key immunological transcription factor, with knockout mice studies identifying functional roles in multiple immune cell types. Despite the importance of NFIL3, little is known about its function in humans.

**Methods:**

Here, we characterised a kindred of two monozygotic twin girls with juvenile idiopathic arthritis at the genetic and immunological level, using whole exome sequencing, single cell sequencing and flow cytometry. Parallel studies were performed in a mouse model.

**Results:**

The patients inherited a novel p.M170I in NFIL3 from each of the parents. The mutant form of NFIL3 demonstrated reduced stability in vitro. The potential contribution of this mutation to arthritis susceptibility was demonstrated through a preclinical model, where Nfil3-deficient mice upregulated IL-1β production, with more severe arthritis symptoms on disease induction. Single cell sequencing of patient blood quantified the transcriptional dysfunctions present across the peripheral immune system, converging on IL-1β as a pivotal cytokine.

**Conclusions:**

NFIL3 mutation can sensitise for arthritis development, in mice and humans, and rewires the innate immune system for IL-1β over-production.

**Key messages**

- Homozygous *NFIL3* mutations identified in monozygotic twins with juvenile idiopathic arthritis.
- Enhanced susceptibility to arthritis induction in *Nfil3*-knockout mice.
- NFIL3 loss in patients and mice is associated with elevated production of IL-1β.
- Knockdown of *NFIL3* in healthy macrophages drives IL-1β production.

## Introduction

Juvenile idiopathic arthritis (JIA) is the most common of the childhood rheumatic diseases. JIA is characterised as juvenile-onset persistent arthritis with no defined cause. A high degree of clinical heterogeneity is observed within the JIA group of diseases, thought to reflect a diversity in genetic and environmental factors and mechanistic drivers. JIA shows similarities to adult autoimmune diseases, and, indeed, genome-wide association studies identify a strong overlap in the common variants linked to autoimmune susceptibility and JIA susceptibility.^1^ JIA also has similarities to autoinflammatory diseases, such as genetic associations to innate inflammatory pathways^2^ and response to IL-1β blockade.^3^ The recent success in identifying monogenic causes of autoinflammatory diseases^4^ suggests that monogenic causes may also underlie a subset of patients with JIA. Indeed, the association of systemic JIA with mutations in *LACCI* 5 supports the potential productivity of this approach in the non-systemic JIA diseases.

NFIL3 is an important transcription factor in the immune system. Analysis of *Nfil3*-deficient mice has identified a key role for Nfil3 in the development of natural killer (NK) cells,^6^ with similar functions in other innate lymphoid cells,^7^ and the CD8α^+^ dendritic cell subset.^8^ Within T cells, Nfil3 enhances the Th2 lineage^9^ while suppressing the Th17 lineage.^10^ The net effect of these changes is the spontaneous development of colitis, a process dependent on microflora and T cell activation.^11^ Less is known about the function of NFIL3 in humans. Expression of NFIL3 is reduced in patients with Crohn’s disease and ulcerative colitis,^12^ and in vitro gene silencing of *NFIL3* in T cells and B cells promotes self-reactivity.^13^ These results suggest that NFIL3 plays a key immune homeostatic role in humans; however, genetically deficient patients are required to understand the in vivo function.

Here, we have characterised monozygotic twins with JIA and inflammatory complications, who harbour homozygous mutations in NFIL3. Parallel studies in mice confirm the arthritogenic potential of NFIL3-deficiency, with *Nfil3* knockout mice showing enhanced susceptibility to arthritis induction. Mechanistic analysis identified elevated production of IL-1β and TNFα by myeloid cells in the peripheral blood of NFIL3 patients and the inflamed joints of *Nfil3*-deficient mice. Our results here demonstrate a link between NFIL3 mutation and restraint of inflammatory cytokine production in the myeloid lineage, contributing to a monogenic form of JIA.

## Methods

### Genetic analysis

The study was approved by the Ethics Committee of UZ Leuven, Belgium, and written informed consent was obtained from the parents of the patients and age-matched healthy individuals. The study was performed in accordance with the modified version of the Helsinki declaration. Whole exome sequencing was performed as previously described on P1 and P2.^14^ Only coding non-synonymous variants with genotype quality >60, gene damage index score of <12 405^15^ and mean allele frequency of <0.005 in NHLBI GO Exome Sequencing Project 6500, 1000 Genomes Project (October 2014) or in The Exome Aggregation Consortium database were considered.

### Human macrophage differentiation

Peripheral blood mononuclear cells (PBMCs) were isolated from heparinised blood using lymphocyte separation medium (LSM, MP Biomedicals). PBMCs were plated in Poly-D-Lysine coated flask in complete RPMI (Thermo Fisher Scientific) and incubated for 2 hours to enrich the monocyte population by plastic adherence. Monocytes were differentiated into macrophages for 7–10 days in differentiation media containing complete RPMI medium supplemented with recombinant hM-CSF (50 ng/mL) and hIL-10 (25 ng/mL) (both BioLegend), replacing media every 2–3 days during the differentiation period. Macrophages were transfected with either scrambled siRNA (SantaCruz, sc-36869, 30 pmol) or three siRNA targeting *Nfil3* mRNA (ThermoFisher assay IDs 144020, 115 656 and 115655, 30 pmol each) by nucleofection (Lonza, VPA-1008, Nucleofector program T-016). Twenty-four hours post-transfection cells were stimulated with 1 µg/mL lipopolysaccharide (LPS) for 24 hours.

### Flow cytometry

PBMCs were isolated using LSM (MP Biomedicals) from patients and healthy individuals. For intracellular staining, cells were plated with complete RPMI containing phorbol myristate acetate (PMA 50 ng/mL; Sigma-Aldrich), ionomycin (500 ng/mL; Sigma-Aldrich) and Brefeldin A (8 ng/mL; Tocris Bioscience) for 4 hours. Cells were fixed and permeabilised with the eBioscience Foxp3 staining kit (eBioscience). Anti-human antibodies included anti-NFIL3 (REA732) (Miltenyi Biotec), anti-CD14 (TuK4) (eBioscience); anti-CD3 (Miltenyi Biotec); anti-CD16 (3G8), anti-CD56 (NCAM16.2), anti-CD123 (7G3), anti-CD27 (L128), anti-CD45RA (HI100), anti-CD8 (SK1), anti-CD4 (SK3), anti-CD1c (L161), anti-IFNγ (4S.B3), anti-T-BET (4B10), anti-IL-17a (N49-653), anti-GATA3 (L50-823) (all from BD Biosciences); anti-HLA-DR (L243), anti-CD19 (HIB19), anti-CD56 (NCAM16.2), anti-CD11c (3.9), anti-CCR7 (G043H7), anti-FOXP3 (206D), anti-RORγt (Q21-559), anti-TNFα (MAb11), anti-IL-4 (MP4-25D2) (all from BioLegend); purified Rabbit-anti-human NFIL3 (D5K8O) (Cell Signaling Technology) followed by Donkey-anti-Rabbit-IgG (Thermo Fisher Scientific). Data were collected on BD Symphony (BD Biosciences) and analysed using FlowJo V.10.5 (Tree Star Inc.).

### Biochemistry

Lysates from lymphoblastoid cells were run on the NuPAGE Precast Gel System (Life Technologies). Thirty to 50 µg of lysate were separated on 4%–12% bis-tris acrylamide gels and blotted on a PVDF membrane (GR Healthcare). Membranes were incubated with rabbit anti-NFIL3 (1:500, D5K80, Cell signaling) and mouse anti-Vinculin (1:2000, V9264, Sigma). Proteins were revealed using western Lightning Prime-ECL (GE Healthcare) and the imaging system G:Box XRQ (Syngene). Quantification was performed using the AIDA software (Raytest, V.5.0).

N-terminally FLAG-tagged human NFIL3-T2A-GFP (WT or carrying the M170I mutation) was expressed transiently from a plasmid in HEK293T. The expression was driven by chicken actin promoter with the CMV enhancer. For transfections, HEK293T cells were grown on poly-L lysine-treated (0.1%) cover slips to subconfluency. Plasmid transfection was done using Lipofectamine 3000 according to the manufacturers protocol (Thermo Fisher). Twenty-four hours after transfection, the cells were washed in PBS, fixed in 4% PFA and permeabilised in 0.1% Triton X-100 (in PBS). After blocking in PBS with 2% bovine serum albumine (BSA), 10% donkey serum and 0.1% Triton X-100 for 30 min, cells were stained with an anti-Flag polyclonal affinity antibody (F7425; Sigma Aldrich) for 2 hours, then washed and incubated for 1 hour with Alexa Fluor 555 donkey-anti-rabbit (A31572; Molecular Probes) antibody as well as DAPI (D1306; Molecular Probes). After washing the cells, they were covered using Fluoromount (Thermo Fisher). Images were collected on an LSM 510 Meta confocal microscope (Ziess) with a 60× immersion objective. Quantification of mean fluorescence intensity was measured using ImageJ software. Alternatively, 24 hours post-transfection, cells were stained with fixable viability dye (eBioscience), fixed and stained for human NFIL3 following the eBioscience protocol for flow cytometry analysis.

### Arthritis induction in mice

C57Bl/6 and *Nfil3* ^-/-^ mice^16^ were bred and housed under barrier conditions at a specific pathogen-free facility at the Walter and Eliza Hall Institute Animal Facility. Eight-to-ten week-old mice were used for all experiments. All procedures were approved by the Walter and Eliza Hall Institute Animal Ethics Committee. Serum transfer arthritis was induced by injection of arthritogenic serum from 12-week-old progeny of KRN and non-obese diabetic mice (K/BxN mice).^17^ Clinical score was assessed as a sum of the clinical score for each paw (0, no erythema and swelling; 1, mild erythema and swelling confined to the ankle, wrist or digits; 2, mild erythema and swelling extending from the ankle to the mid-foot; 3, moderate erythema and swelling extending from the ankle to the metatarsal joints; 4, severe erythema and swelling extending the entire limb and with joint ankylosis). The severity of joint inflammation was also assessed with in vivo imaging of bioluminescence using luminol, a substrate for myeloperoxidase activity (in myeloid cells), on days 4 and 7, as published previously.^18^ Arthritis of the ankle joint was evaluated histologically from two independent experiments. Front and hind limbs of mice were fixed in 10% neutral-buffered formalin, embedded in paraffin, sectioned at 7 µm and stained with Safranin-O, according to standard practices. Histological analysis was performed on serial joint sections. Histology scores are as follows: 0=normal, 1=moderate, 3=severe.

Flow cytometry was performed on cells isolated from the peritoneal lavage, joints and blood. For cytokine production measurement, cells were stimulated with LPS (0.1 µg/mL) in the presence of Brefeldin A and monensin for 3 hours, stained for surface markers, followed by intracellular staining of IL-1β and TNF.

### Single cell sequencing

Peripheral blood was collected by venipuncture, and the PBMC fraction was isolated using LSM-Lymphocyte Separation Medium (MP Biomedicals). PBMC were then viably frozen and stored in liquid nitrogen prior to single cell sequencing. On thawing, the PBMC were counted using a Countess II Automated Cell Counter (Thermo Fisher), and 8700 cells for each sample were loaded individually onto the Chromium Controller (10x Genomics).

### Analysis of single-cell RNA-seq data from patient and control PBMCs

Sequence data were preprocessed with Cell Ranger V.2.0 (10x Genomics). The resulting count matrices were analysed with R V.3.4 and the package Seurat V.2.2 (6), following the standard pipeline with default parameters, unless stated otherwise. Genes detected in less than five cells as well as cells with less than 500 genes detected were filtered out, leaving 15 216 genes across 4743 cells in the control and 14 367 genes across 2165 cells in the patient. Gene expression was normalised across genes by dividing by the total expression per cell, log-transformed and standardised across cells. The 1000 most variable genes were used to align the expression levels of both samples, through the components of a canonical correlation analysis (CCA). The tSNE plots were calculated on the first 20 components of the CCA, and clusters were identified by the community-detection algorithm implemented by Seurat.

Gene set enrichment analysis was carried out for each cluster (cell type) with GSEA v 3.0 (Broad Institute, Cambridge, Massachusetts, USA) (7). Gene sets with size larger than 1000 or smaller than 10 were excluded. Detection of variation in gene sets was controlled to have a false discovery rate lower than 0.25. Gene sets were prioritised according to the normalised enrichment score provided by GSEA.

KEGG pathways^19^ were analysed with Pathview,^20^ through the web server API. First, each cluster (cell type) was analysed separately, using all genes with detected fold-change, for the pathways corresponding to signal transduction, immune system and immune diseases. Then, the final pathway representation was obtained by merging the expression levels of the genes directly related to each cell type.

### Real time PCR

RNA has been isolated from sorted CD14+ monocytes, differentiated macrophages or NIH3T3 cells using the ReliaPrep RNA Cell Miniprep System (Promega). cDNA synthesis was performed using the Superscript III RT System (Thermos Fisher). Expression of *STX11, TGFB1, CSF2RB, CEBPG, CD224, NFIL3, TNF, IL1B, HPRT, RPL0, ACTB* and plasmid-encoded ncRNA was measured by PrimeTime qPCR Probe Assays (IDT) and IL1B by SYBR green qPCR (Thermo Fisher). The expression of *HPRT, RPL0* and *ACTB* was used to normalise mRNA expression.

## Results

### NFIL3 mutations in monozygotic twins with juvenile idiopathic arthritis

**Figure 1:**
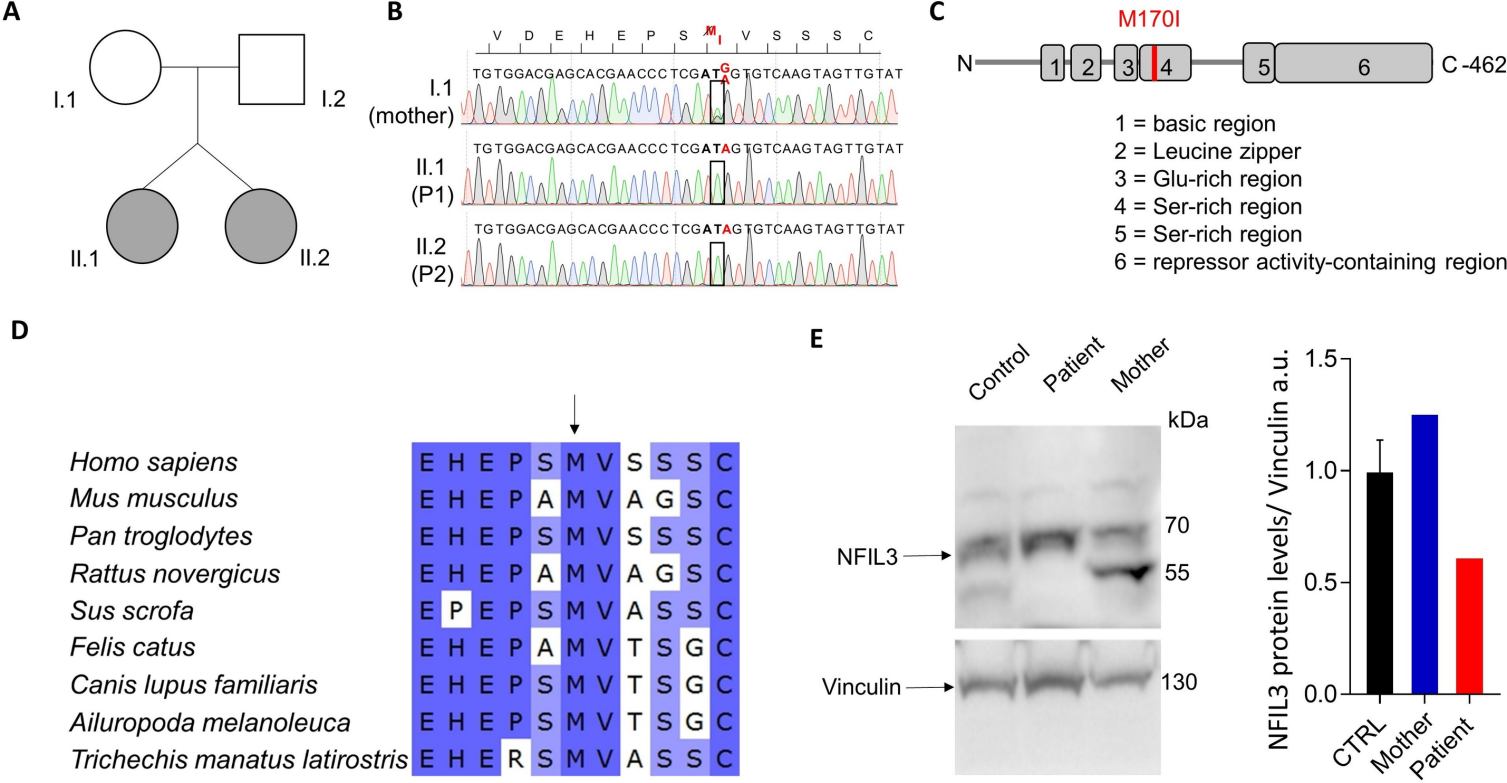
NFIL3 mutations in a pedigree with juvenile idiopathic arthritis. (A) Family pedigree of the affected patients (grey). (B) Sanger sequencing of *NFIL3* indicating the site of mutation. (C) Schematic of NFIL3 domains and the site of mutation. (D) Cross-species conservation of NFIL3 in the region flanking M170 (ClustalW). Amino acids with >50% conservation are indicated in blue. (E) Western blot indicating protein expression of NFIL3 in LCLs from control individuals, the patient (homozygous) and mother (heterozygous), with quantification normalised against vinculin.

Monozygotic twins were identified with JIA (figure 1A). Both sisters were diagnosed with oligoarticular JIA at the age of 4 years (P1) and 6 years (P2), respectively. Systemic inflammation at onset (sedimentation 49;<20 mm/hour, CRP 15.2; <5 mg/L, IgG 14.8; 4.78–11.29 g/L (P1); sedimentation 32; <20 mm/hour, CRP 2.9; IgG 17.50; 5.58–12.54 g/L (P2)) and antinuclear antibodies (table 1) were present in both. There was no occurrence of uveitis. Autoimmune thyroiditis developed at age 9 years (P1) and 11 years (P2). P1 was initially treated with intra-articular steroids and methotrexate, with adalimumab added after a relapse. At 11 years of age, laboratory tests revealed mildly increased liver enzymes with normal bilirubin levels, normal NSE, αFP and coagulation tests. Liver ultrasonography with duplex Doppler showed a large well-marginated lesion in the left liver lobe displacing the left subhepatic vein, with a characteristic spoke wheel vascularisation pattern compatible with focal nodular hyperplasia. MRI confirmed a T2 isointense multilobulate tumour with a central T2 hyperintense scar, occupying the left liver lobe. Methotrexate and adalimumab were stopped, with subsequent normalisation of liver enzymes. Over the course of the following 2 years, the hepatic lesion has remained stable but she recently suffered another relapse of knee arthritis. P2 remains in clinical remission after a course of non-steroidal anti-inflammatory drugs and an intra-articular steroid infiltration. The rare occurrence of JIA and autoimmune thyroiditis in monozygotic twins with a family history of psoriatic arthritis in the maternal grandmother, suggested a genetic driver of disease. The co-occurrence of focal nodular hyperplasia of the liver, not previously reported in patients with JIA, was also indicative of a systemic immune defect.

**Table 1:** Patient autoantibody characteristics

	P1	P2
ANA (titre)	Positive (1/640)	Positive (1/320)
Description	Homogenous and chromosomal straining pattern in the nucleus, cytoplasm negative	Homogenous and chromosomal straining pattern in the nucleus, cytoplasm negative
Anti-DNA Farr (cut-off)	7.1 IU/mL (≥7.0)	9.1 IU/mL (≥7.0)
CTD screening*	Negative	Negative
p-ANCA (titre)	1/320	1/160
MPO-ANCA	Negative	Negative
PR3-ANCA	Negative	Negative
Thyroglobulin Ab (cut-off)	385 IU/mL (≥115)	139 IU/mL (≥115)
Thyroid peroxidase Ab (cut-off)	63 IU/mL (≥34)	154 IU/mL (≥34)
IgG (normal range)	15.9 g/L (5.58–12.54)	17.5 g/L (5.58–12.54)
HLA-B27	Positive	Positive
HLA-B51	Negative	Negative

*CTD (connective tissue disease) screening covers SSB/La, U1-RNP, RNP-70, SmD, Scl-70, Jo-1 and Ro60 antigens.

ANA, anti-nuclear antibodies; ANCA, anti-neutrophil cytoplasmic antibodies; CTD, connective tissue disease; HLA-B, human leucocyte antigen; MPO-ANCA, myeloperoxydase anti-neutrophil cytoplasmic antibodies; PR3-ANCA, proteinase 3 anti-neutrophil cytoplasmic antibodies.

Genetic analysis of the patients through whole exome sequencing identified a homozygous mutation in *NFIL3*. Genetic variants were filtered for rare coding mutations. Based on the family history, recessive inheritance was deemed most likely. The patients were found to harbour one rare coding mutation in homozygosity, a G510A mutation in *NFIL3*, resulting in a methionine to isoleucine mutation at residue 170 (M170I). The mutation was confirmed by Sanger sequencing as homozygous in the affected patients and heterozygous in the parents (figure 1B). The mutation is in the Ser-rich region (figure 1C), in a highly conserved stretch of amino acids (figure 1D). Patient cell lines demonstrated a ~50% reduction NFIL3 expression at the protein level (figure 1E). In ex vivo primary cells, taken from an inflammatory environment, NFIL3 mRNA was increased; however, a ~50% reduction in the mRNA/protein ratio was observed (online supplementary figure S1). To formally test protein stability, we transfected cell lines with either the wildtype or M170I form of NFIL3 and observed 50% lower expression of the M170I allele (online supplementary figure S2). Together, these results indicate that M170I NFIL3 is unstable, without excluding additional functional loss from the amino acid change.

10.1136/annrheumdis-2018-213764.supp1 Supplementary data

### NFIL3 knockout mice have enhanced susceptibility to arthritis induction

**Figure 2:**
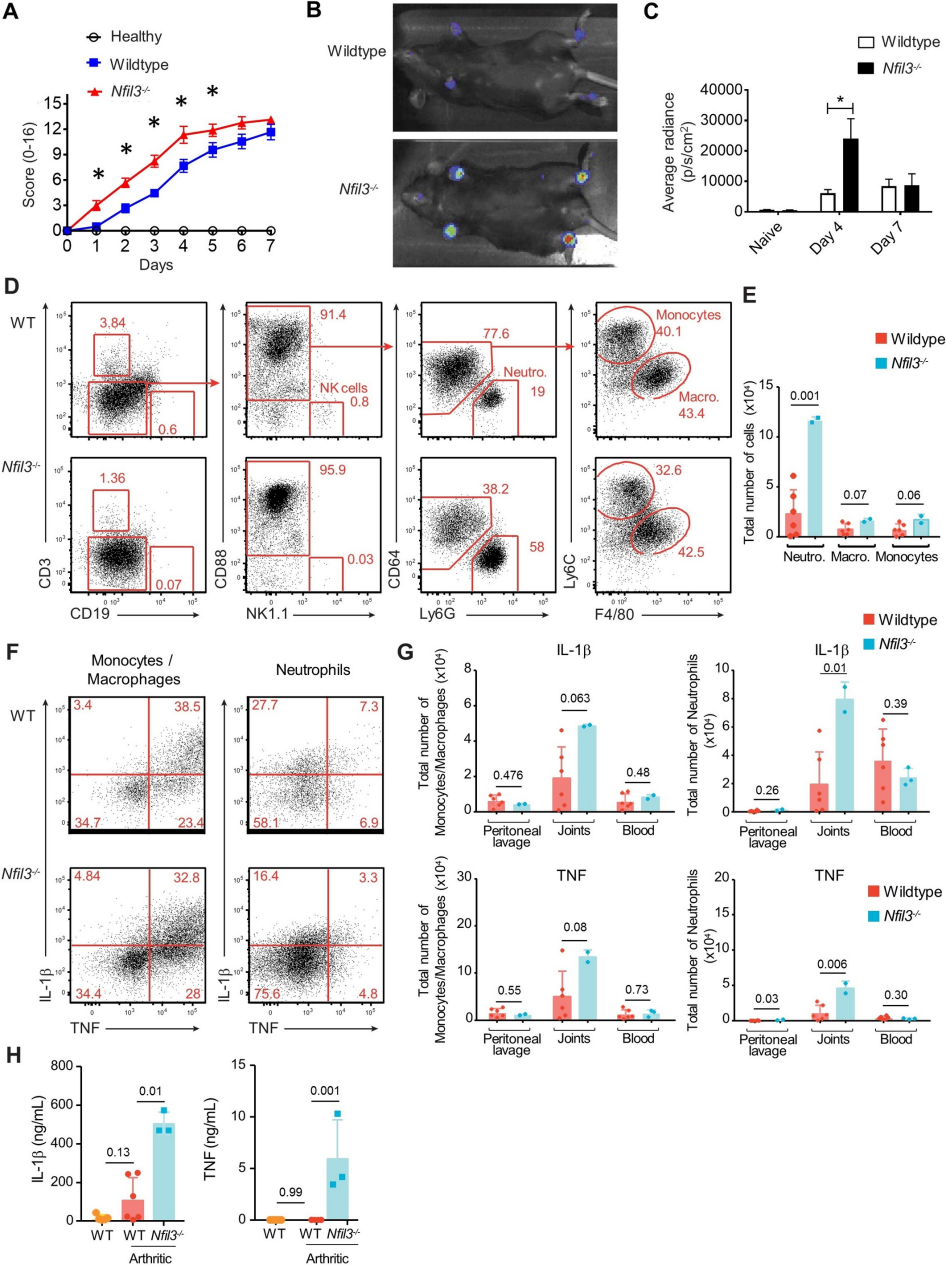
*Nfil3* *^-/-^* mice have increased susceptibility to arthritis induction.﻿ Wildtype and *Nfil3^-/-^* mice were injected with serum from K/BxN mice. (A) Mice were scored for clinical arthritis daily for 7 days. Each paw was scored on a scale of 0–4 based on signs of swelling and inflammation (n=9/group). (B) Mice were imaged for MPO activity in paws using luminol sodium salt solution and were imaged for bioluminescence using the IVIS spectrum imaging. Representative picture and (C) average RADIANCE at days 4 and 7. (D) Wild-type and *Nfil3^-/-^* mice were assessed by flow cytometry 5 days after injection of K/BxN serum. Data are representative of two independent experiments with 6 wild-type and 2–3 *Nfil3^-/-^* mice per experiment. Representative gating of neutrophils, macrophages and monocytes, and (E) quantification of joint-infiltrating cells. (F) Representative flow cytometry analysis showing the intracellular expression of IL-1β and TNF in monocytes and macrophages (CD88^+^Ly6G^-^CD64^+^) and neutrophils (CD88^+^Ly6G^+^CD64^-^) from joints of wild-type and *Nfil3^-/-^* mice. (G) Total numbers of IL-1β-producing and TNF-producing leucocytes are shown from wild-type and *Nfil3^-/-^* in peritoneal lavage, joints and blood. (H) Concentrations of IL-1β and TNF were determined from joint lavage of mice 5 days after injection of K/BxN serum by ELISA. Mean±SD, *p<0.05. MPO, myeloperoxidase.

In the absence of a second family with NFIL3 mutations, we turned to a mouse model. *Nfil3* knockout mice have been previously characterised as possessing a diverse set of immunological alterations.^6–11^ Here, we challenged 8–10-week-old C57BL/6 and *Nfil3* gene deleted mice with arthritogenic serum antibodies derived from the K/BxN mouse strain. This model bypasses early priming stages and compares sensitivity to downstream arthritis pathology processes. Compared with wildtype mice, *Nfil3* knockout mice developed inflammatory arthritis earlier and had more severe joint inflammation, as assessed clinically (figure 2A), by in vivo imaging figure 2A-C and histologically (online supplementary figure S3). Investigation of the inflamed joints of wildtype and *Nfil3* knockout mice identified an elevated myeloid infiltrate, dominated by neutrophils (figure 2D,E). Infiltrating neutrophils and monocytes/macrophages demonstrated enhanced production of IL-1β and TNF in the *Nfil3* knockout joint (figure 2F,G). These changes in the joint were reflected in the serum, with elevated IL-1β and TNF in the arthritic *Nfil3* knockout mice (figure 2H). Together, these results support NFIL3 as a genetic contributor to inflammatory arthritis in the patient pedigree and identify innate inflammatory cytokines as a potential mechanism.

### NFIL3 mutations drive elevated IL-1β production in myeloid cells

**Figure 3:**
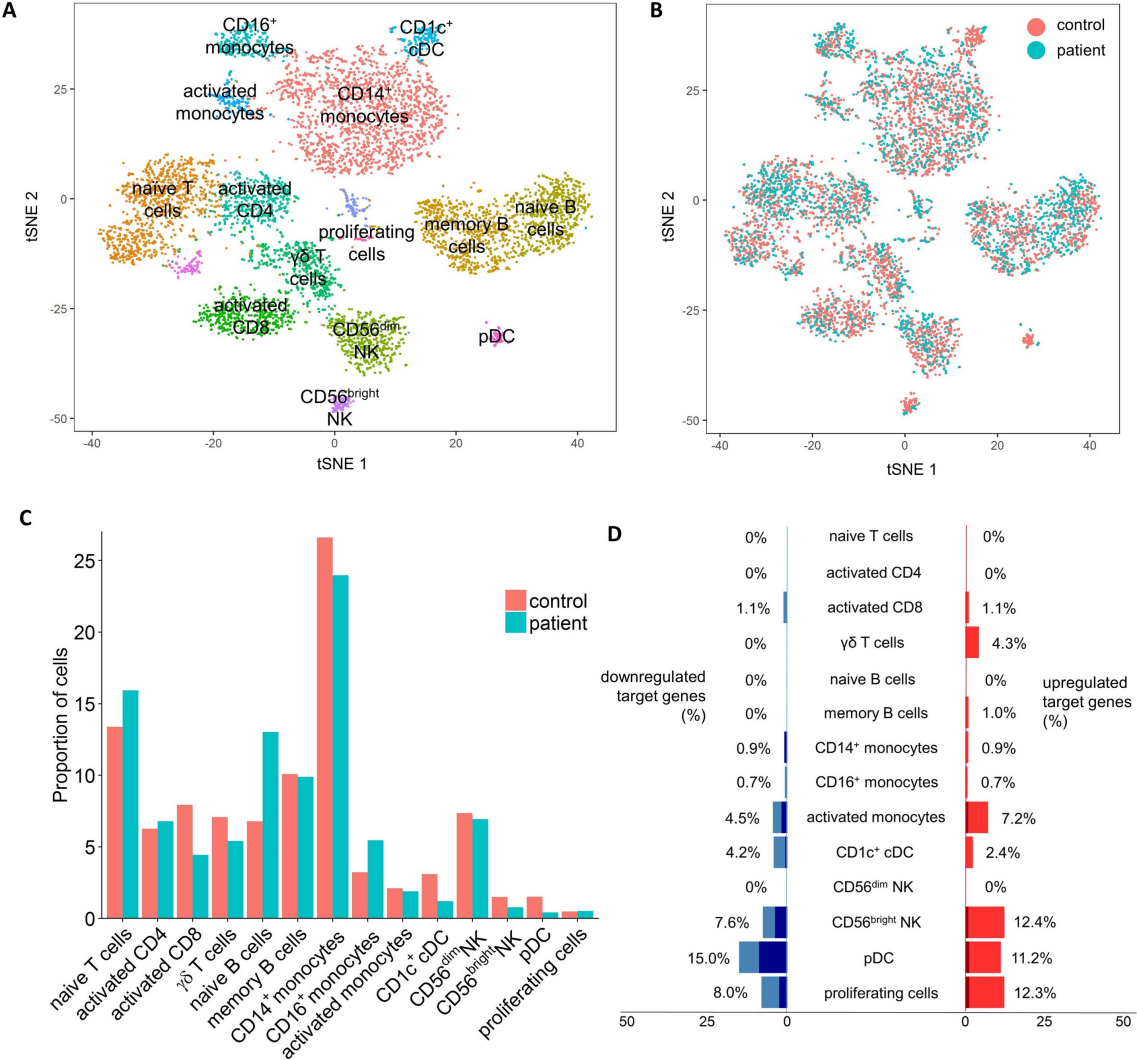
Peripheral immune alterations with NFIL3 mutation. Integrated analysis of single cell sequencing transcriptomics data from patient and control PBMCs. (A) tSNE projection of 6908 PBMCs. After alignment, each cell is grouped into clusters (distinguished by colour). Single joint clustering revealed 14 immune populations annotated according to the expression of key lineage markers. (B) tSNE projection of 6908 PBMCs, split between patient and control after alignment. (C) Proportion of the total number of cells from each sample belonging to each leucocyte population. (D) Proportion of known NFIL3 target genes with a 2-fold (light blue/light red) or 4-fold (dark blue/dark red) expression change, within each leucocyte cluster. Only NFIL3 targets expressed within the cluster were considered. PBMCs, peripheral blood mononuclear cells.

In order to determine the immunological impact of NFIL3 loss of function on the peripheral immune system, we ran a single cell sequencing experiment on P1 and a healthy age-matched control. After data curation, data from 4743 cells from the healthy individual and 2165 cells from the patient were clustered using a tSNE approach (figure 3A). Clusters were manually annotated into leucocyte populations based on the expression of key lineage markers (online supplementary figure S4). Quantification of the clustered leucocyte populations revealed multiple immunological abnormalities in the patient (figure 3B,C). The adaptive immune system gave indications of defective activation, with increased naïve B cells and T cells, while memory B cells and activated T cells were normal and activated CD8 T cells were reduced. Changes were also observed in the innate immune system, with a shift from the CD14^+^ monocyte cluster to the CD16^+^ monocyte cluster, a relative defect in the CD56^bright^ NK cluster and reduced frequencies of both CD1^+^ DC and pDC (figure 3C).

**Figure 4:**
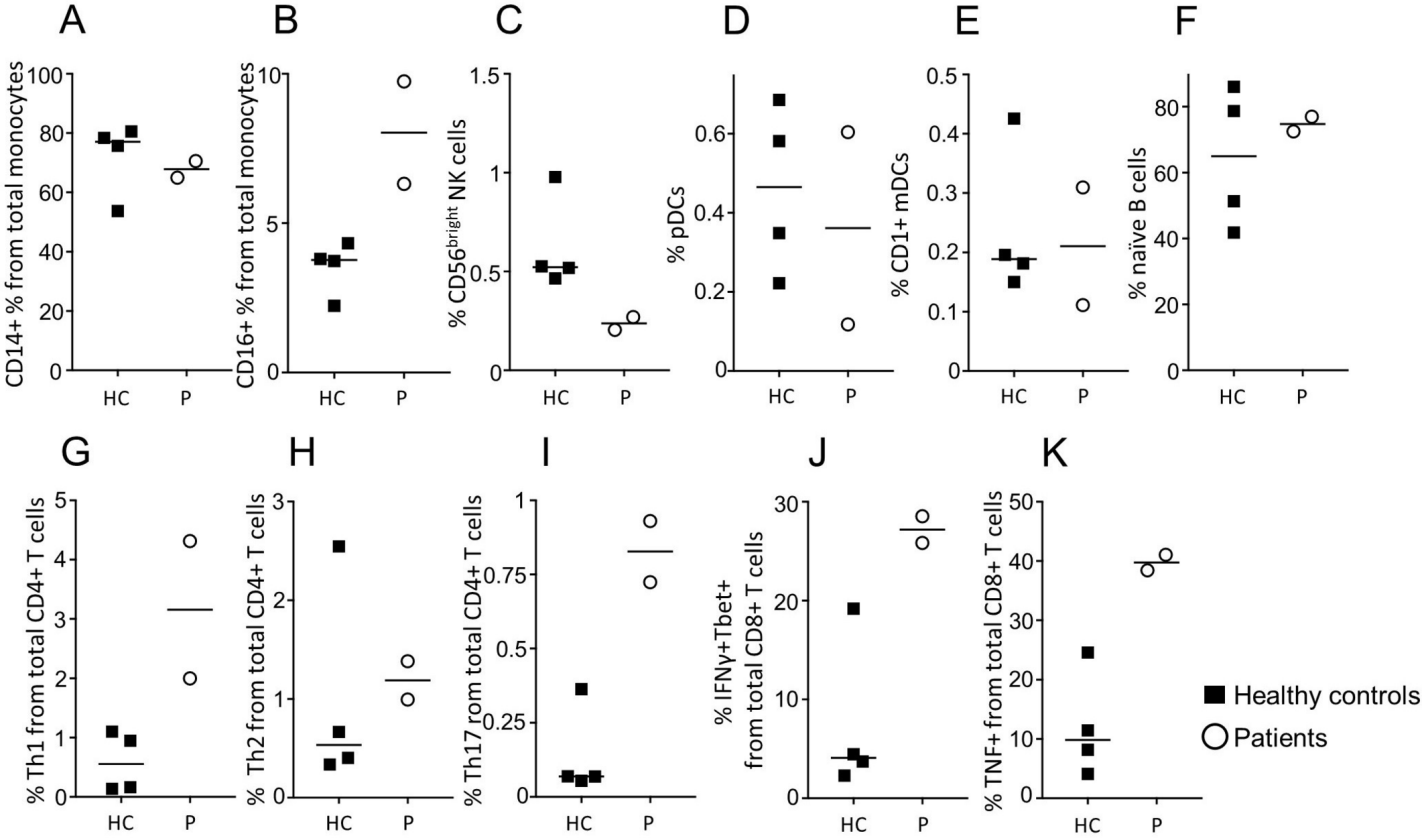
Distinct immunological profiles of patient peripheral blood. Peripheral blood from healthy controls (black squares) and the two patients (open circles) were assessed for immune phenotype by flow cytometry. (A) CD14^+^ monocytes (CD14^+^CD16^-^HLADR^+^), (B) CD16^+^ monocytes (CD16^+^CD14^-^HLADR^+^), (C) CD56^bright^ NK cells (CD3^-^CD19^-^CD14^-^CD16^-^CD56^bright^), (D) plasmacytoid DCs (CD3^-^CD19^-^CD14^-^CD56^-^HLADR^+^CD11c^low^CD123^+^), (E) CD1c^+^ myeloid DCs (CD3^-^CD19^-^CD14^-^CD56^-^HLADR^+^CD11c^+^CD1c^+^CD123^-^), (F) naïve B cells (CD19^+^CD14^-^CD27^-^), (G) Th1 (CD3^+^CD4^+^IFNγ^+^TBET^+^), (H) Th2 (CD3^+^CD4^+^IL4^+^GATA3^+^), (I) Th17 (CD3^+^CD4^+^RORγ^+^IL17^+^), (J) CD3^+^CD8^+^IFNγ^+^TBET^+^, (K) CD3^+^CD8^+^TNFα^+^. Median and individual data points are shown.

To validate the changes observed using single cell sequencing, we used a flow cytometric analysis on both P1 and P2 and four healthy controls (figure 4). As was observed using single cell sequencing, the innate immune system was disturbed in the NFIL3 patients, with an increased frequency of CD16^+^ monocytes (figure 4B) and a selective reduction in the CD56^bright^ NK population (figure 4C). Analysis of T cell populations with flow cytometry picked up an increase in T cell activation not apparent at the transcript level. Th1, Th17, IFNγ-producing CD8 and TNF-producing CD8 T cells were all increased (figure 4G,I–K). These results validated and extended the single cell analysis, identifying an inflammatory milieu in NFIL3 patients.

**Figure 5:**
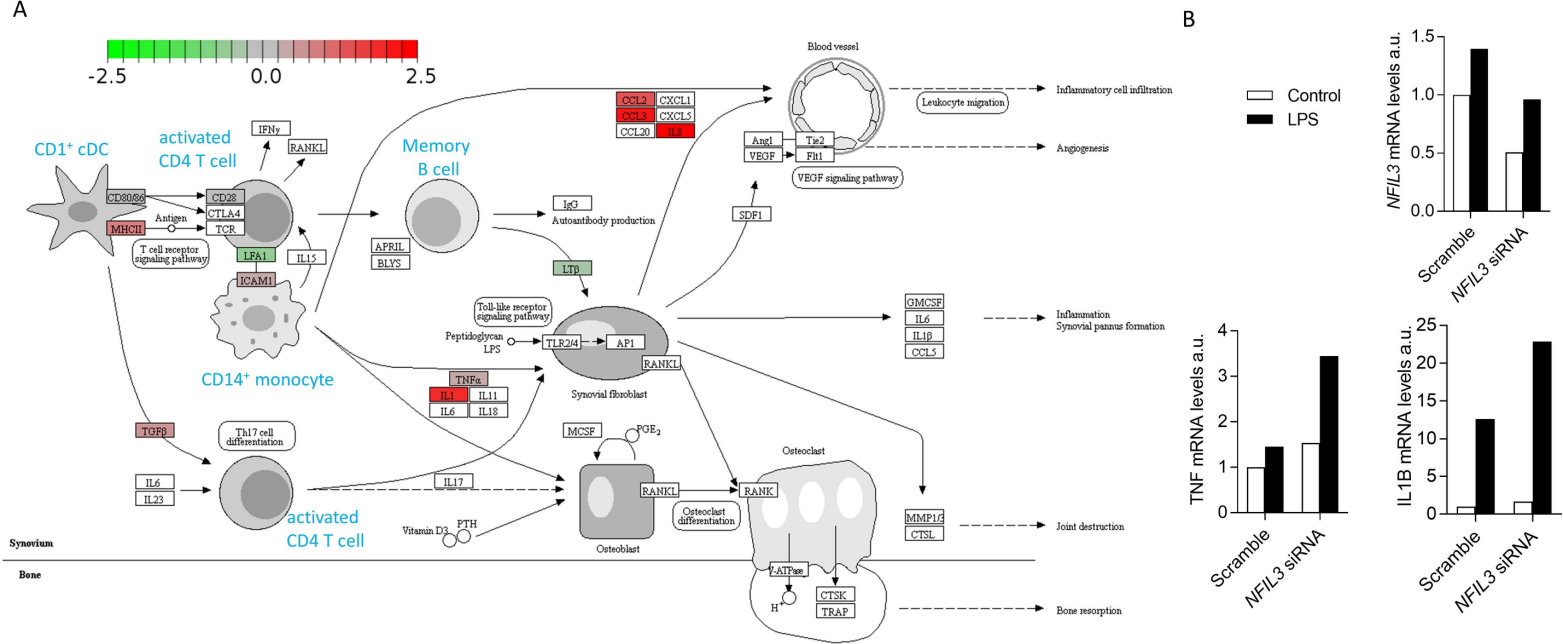
Mapping of transcriptional changes in NFIL3 patient onto arthritogenic pathways. (A)﻿ Single cell sequencing transcriptomics data from patient and control PBMCs was mapped onto KEGG pathways. Transcriptional changes in the KEGG rheumatoid arthritis pathway were visualised using an adapted Pathview. In blue are shown labels for mapped cell types, corresponding to annotated single cell clusters. Differential gene expression within each annotated cell type is visualised with colour, with green indicating overexpression in healthy control and red indicating overexpression in patient. Synovial stromal cells, not present in the single cell RNAseq dataset, are represented but with annotated genes indicated as transcript not detected (white). (B) Healthy control PBMCs were differentiated into macrophages and treated with either scrambled siRNA or NFIL3 siRNA, and NFIL3 mRNA knockdown was confirmed by qPCR. Treated macrophages were stimulated with LPS for 24 hours, following which IL1β and TNFα mRNA expression was assessed by qPCR. PBMCs, peripheral blood mononuclear cells.

Beyond changes in leucocyte population frequency, we used the single cell data to detect altered transcriptional pathways in NFIL3 patients. Global transcriptional analysis indicated differential effects of NFIL3 deficiency of each population (online supplementary figure S6). Naïve T cells and CD56^dim^ NK cells were dominated by an upregulation of ribosomal components and protein production machinery. By contrast, activated T cells and B cells demonstrated increased expression of multiple transcription factors, including FOS, MYC, IRF1 and STAT3 (online supplementary figure S6), indicating stronger levels of activation. Many biological pathways were altered in the myeloid compartment, with the upregulation of components of the MAPK pathway the key feature (online supplementary figure S6), again indicative of excessive activation. When transcriptional changes were mapped onto the Rheumatoid Arthritis KEGG pathway, excessive production of IL-1β and TNF by innate leucocytes was identified as a key change (figure 5A), corresponding with the changes observed in mice (figure 2). Due to the known arthritogenic role of IL-1β, we tested whether a direct link could be established between NFIL3 expression in macrophages and IL-1β production. Using an siRNA approach, we knocked down *NFIL3* expression in primary macrophages cultured from a healthy individual and found that ~50% reduction in NFIL3 primed macrophages for excessive IL-1β and TNF expression (figure 5B). This mechanistic analysis suggests that the effects of NFIL3-deficiency may be pleiotropic, with differential rewiring of multiple leucocyte populations culminating in dysregulated IL-1β and TNF production in an arthritogenic reaction.

## Discussion

In this study, the in vivo immunological role of NFIL3 has been characterised, with deficiency in NFIL3 sensitising to arthritis development in mice and in patients. Mechanistic analysis in both species converged on IL-1β overproduction by innate leucocytes as a potential disease mechanism. It is likely, however, that the effect of NFIL3 is more pleiotropic, with multiple complex interactions. For example, the adaptive immune system in these patients also demonstrated a Th1/Th17 skew, which may also contribute to disease. A proinflammatory phenotype of NFIL3 deficiency is consistent with both the murine model, which develops colitis,^11^ and correlative data in humans, where NFIL3 expression is reduced in patients with colitis.^12^ While the patients described here have not presented with colitis, it is increasingly recognised that the clinical presentation of autoinflammatory diseases is diverse, with the underlying biological defect manifesting as different clinical symptoms in different individuals. The identification of *NFIL3* as an autoinflammatory gene opens up further investigation of monogenic patients, who may present with inflammatory phenotypes across the spectrum.

Independent of the role of *NFIL3* mutations in disease, the identification of an NFIL3-deficient family allows the first analysis of the in vivo functions of NFIL3 in humans. In vitro gene silencing on NFIL3 in human T cells and B cells has been performed;^13^ however, in vivo experiments on NFIL3-deficiency have been restricted to mice. Comparison of the NFIL3-deficient patients assessed here with the Nfil3-deficient mice reveals both cross-species similarities and species-specific functions. The patients, as with the mice,^6^ have defects in NK cells, with a reduction in maturation to the CD56^bright^ population. Likewise, in patients, NFIL3 deficiency results in a major loss of the cDC population, phenocopying mice.^8^ Here, we demonstrated a mechanistic link between NFIL3 expression and proinflammatory cytokine production, and an association between NFIL3 deficiency with arthritis in mice and patients.

## References

[1] McIntosh LA, Marion MC, Sudman M, et al Genome-Wide Association Meta-Analysis Reveals Novel Juvenile Idiopathic Arthritis Susceptibility Loci. Arthritis Rheumatol 2017;69:2222–32. 10.1002/art.40216 28719732 PMC5874801

[2] Hinks A, Martin P, Thompson SD, et al Autoinflammatory gene polymorphisms and susceptibility to UK juvenile idiopathic arthritis. Pediatr Rheumatol Online J 2013;11:14 10.1186/1546-0096-11-14 23547563 PMC3621775

[3] Brachat AH, Grom AA, Wulffraat N, et al Early changes in gene expression and inflammatory proteins in systemic juvenile idiopathic arthritis patients on canakinumab therapy. Arthritis Res Ther 2017;19:13 10.1186/s13075-016-1212-x 28115015 PMC5260050

[4] Manthiram K, Zhou Q, Aksentijevich I, et al The monogenic autoinflammatory diseases define new pathways in human innate immunity and inflammation. Nat Immunol 2017;18:832–42. 10.1038/ni.3777 28722725

[5] Wakil SM, Monies DM, Abouelhoda M, et al Association of a mutation in LACC1 with a monogenic form of systemic juvenile idiopathic arthritis. Arthritis Rheumatol 2015;67:288–95. 10.1002/art.38877 25220867

[6] Kamizono S, Duncan GS, Seidel MG, et al Nfil3/E4bp4 is required for the development and maturation of NK cells in vivo. J Exp Med 2009;206:2977–86. 10.1084/jem.20092176 19995955 PMC2806474

[7] Seillet C, Rankin LC, Groom JR, et al Nfil3 is required for the development of all innate lymphoid cell subsets. J Exp Med 2014;211:1733–40. 10.1084/jem.20140145 25092873 PMC4144736

[8] Kashiwada M, Pham NL, Pewe LL, et al NFIL3/E4BP4 is a key transcription factor for CD8α⁺ dendritic cell development. Blood 2011;117:6193–7. 10.1182/blood-2010-07-295873 21474667 PMC3122942

[9] Motomura Y, Kitamura H, Hijikata A, et al The transcription factor E4BP4 regulates the production of IL-10 and IL-13 in CD4+ T cells. Nat Immunol 2011;12:450–9. 10.1038/ni.2020 21460847 PMC3494493

[10] Yu X, Rollins D, Ruhn KA, et al TH17 cell differentiation is regulated by the circadian clock. Science 2013;342:727–30. 10.1126/science.1243884 24202171 PMC4165400

[11] Kobayashi T, Steinbach EC, Russo SM, et al NFIL3-deficient mice develop microbiota-dependent, IL-12/23-driven spontaneous colitis. J Immunol 2014;192:1918–27. 10.4049/jimmunol.1301819 24442434 PMC3948213

[12] Kobayashi T, Matsuoka K, Sheikh SZ, et al NFIL3 is a regulator of IL-12 p40 in macrophages and mucosal immunity. J Immunol 2011;186:4649–55. 10.4049/jimmunol.1003888 21383239 PMC3172700

[13] Zhao M, Liu Q, Liang G, et al E4BP4 overexpression: a protective mechanism in CD4+ T cells from SLE patients. J Autoimmun 2013;41:152–60. 10.1016/j.jaut.2013.01.004 23340290

[14] Masters SL, Lagou V, Jéru I, et al Familial autoinflammation with neutrophilic dermatosis reveals a regulatory mechanism of pyrin activation. Sci Transl Med 2016;8:332ra45 10.1126/scitranslmed.aaf1471 27030597

[15] Itan Y, Shang L, Boisson B, et al The human gene damage index as a gene-level approach to prioritizing exome variants. Proc Natl Acad Sci U S A 2015;112:13615–20. 10.1073/pnas.1518646112 26483451 PMC4640721

[16] Gascoyne DM, Long E, Veiga-Fernandes H, et al The basic leucine zipper transcription factor E4BP4 is essential for natural killer cell development. Nat Immunol 2009;10:1118–24. 10.1038/ni.1787 19749763

[17] Monach PA, Mathis D, Benoist C The K/BxN arthritis model. Curr Protoc Immunol 2008;Chapter 15:Unit 15.22 10.1002/0471142735.im1522s81 18491295

[18] Campbell IK, Leong D, Edwards KM, et al Therapeutic Targeting of the G-CSF Receptor Reduces Neutrophil Trafficking and Joint Inflammation in Antibody-Mediated Inflammatory Arthritis. J Immunol 2016;197:4392–402. 10.4049/jimmunol.1600121 27807194

[19] Kanehisa M, Furumichi M, Tanabe M, et al KEGG: new perspectives on genomes, pathways, diseases and drugs. Nucleic Acids Res 2017;45(D1):D353–D361. 10.1093/nar/gkw1092 27899662 PMC5210567

[20] Luo W, Pant G, Bhavnasi YK, et al Pathview Web: user friendly pathway visualization and data integration. Nucleic Acids Res 2017;45(W1):W501–W508. 10.1093/nar/gkx372 28482075 PMC5570256

