# Supplementary material for: NFIL3 mutations alter immune homeostasis and sensitise for arthritis pathology
Source: Ann Rheum Dis. 2018 Dec 14;78(3):342–9. doi: 10.1136/annrheumdis-2018-213764 (PMC6390028; doi:10.1136/annrheumdis-2018-213764)
Supplement: Supplementary data [file annrheumdis-2018-213764supp001.pdf]

Supplementary figures

Figure S1. Reduced mRNA/protein ratio in M170I NFIL3 patients. (A) $CD14^+$ monocytes and $CD16^+$ monocytes were sorted from controls and patients, prior to NFIL3 qPCR. **(B)** NFIL3 expression was assessed on $CD14^+$ monocytes and $CD16^+$ monocytes by flow cytometry. **(C)** The ratio of protein/mRNA was calculated for patients and healthy controls using the qPCR and flow cytometry data. Mean and individual values shown.

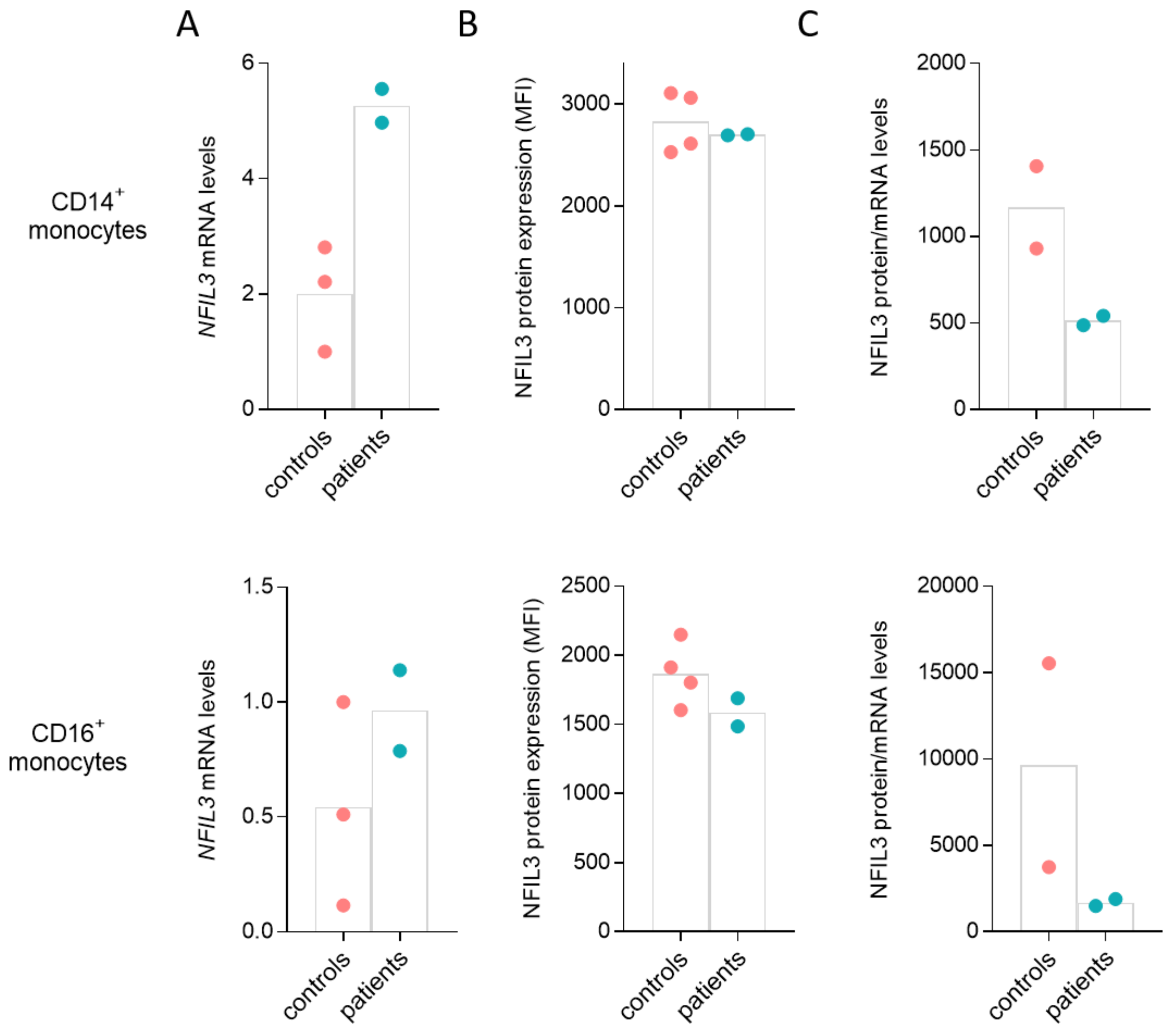

Figure S2. M170I mutation in NFIL3 causes decreased protein stability. HEK293T cells were transfected with FLAG-tagged NFIL3-GFP, using either the wildtype or M170I mutant form. **(A)** Transfected cells were imaged with DAPI and anti-FLAG antibodies, with representative images of wildtype NFIL3 and **(B)** M170I NFIL3 transfections. Scale bar = 50µm. **(C)** Quantification of FLAG-NFIL3 immunofluroscence in wildtype and mutant transfected cells (n=92,104 cells). Mean±Standard deviation. **(D)** Transfected cells were assessed for plasmid copy number, using qPCR quantification of a plasmid-encoded ncRNA. **(E)** Transfected cells were assessed for NFIL3 mRNA expression using qPCR quantification and **(F)** NFIL3 protein expression, using flow cytometry. Mean and individual values shown.

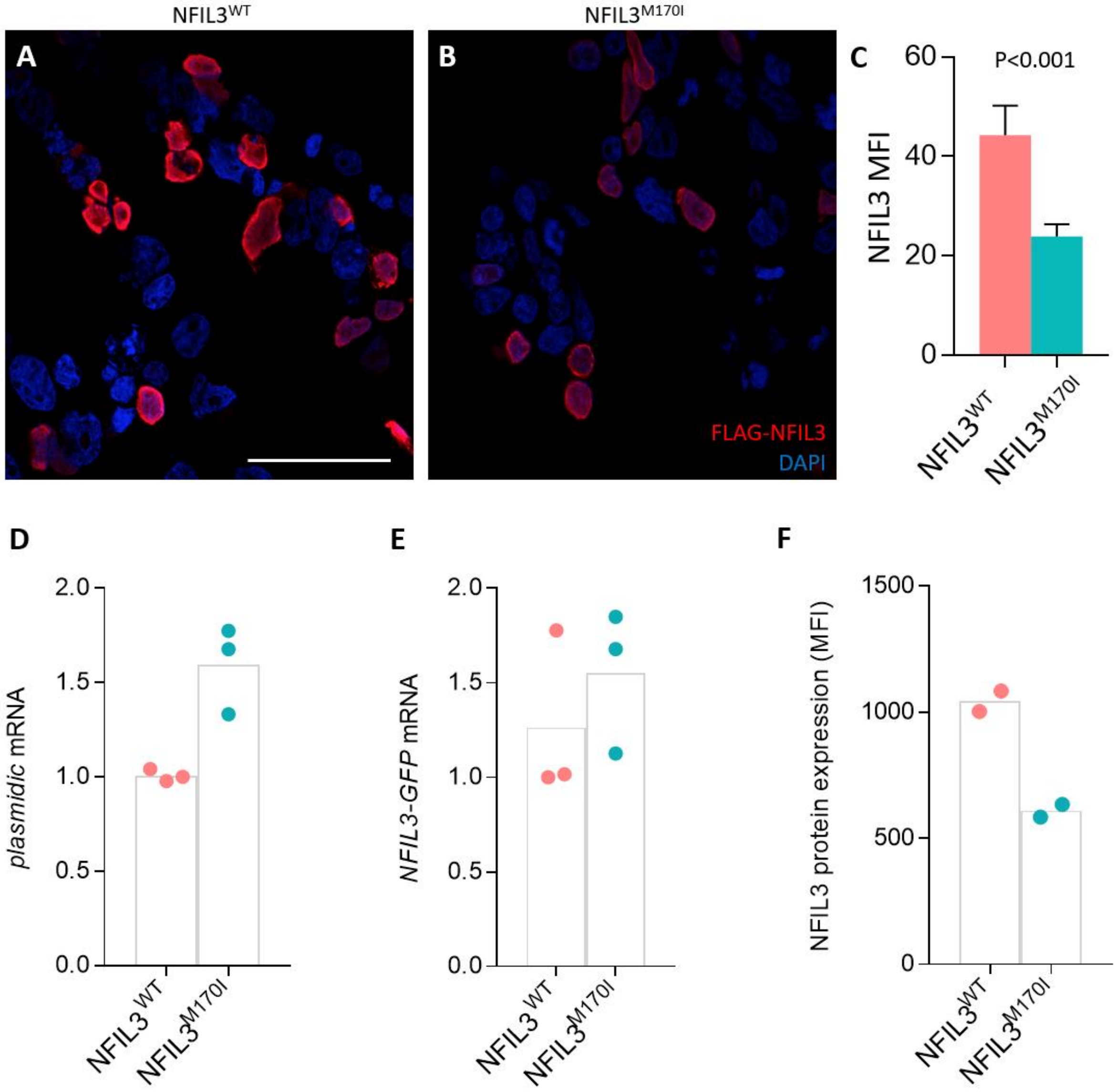

Figure S3. Enhanced histological damage following arthritis induction in NFIL3 knockout mice. Wildtype and *Nfil3*$^{-/-}$ mice were injected with serum from K/BxN mice (n=9/group). Mice were scored for histological damage on day 7. Mean±SD; * $p<0.05$.

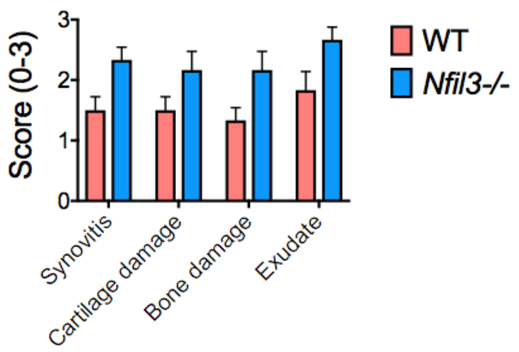

Figure S4. Visualization of expression of specific lineage markers (colored single cells) on tSNE plot projecting 6,908 PBMCs from patient and control.

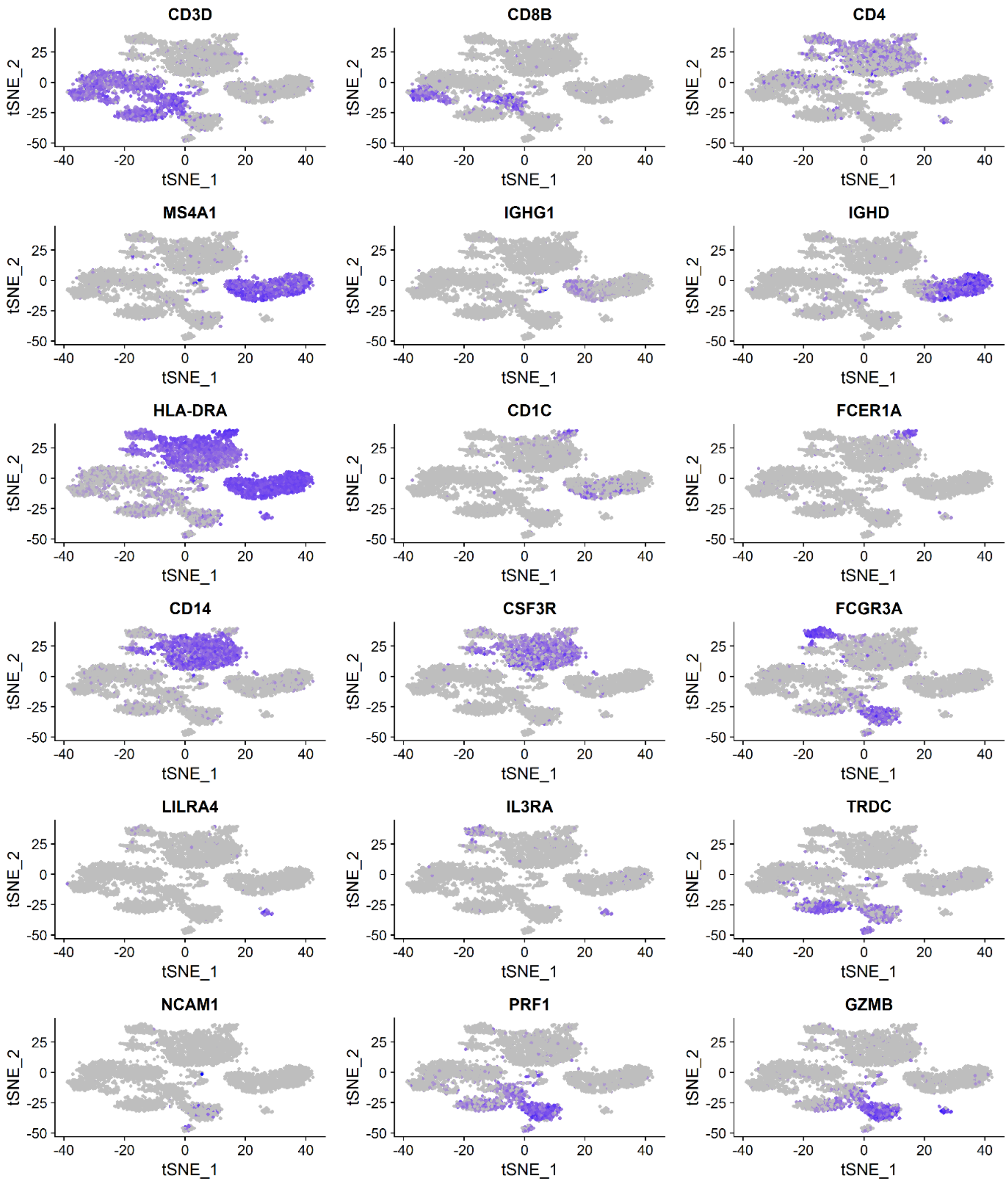

Figure S5. Validation of single cell RNAseq gene expression changes. CD14^{+} monocytes were sorted from P1 and age-matched gender-matched control. **(A)** Expression of NFIL3 target genes *STX11*, *TGFB1*, *CSF2RB*, *CEBPG*, *CD224* normalized to *HPRT*, *RPL0*, *ACTB* mRNA. Patient (P1) and age and gender matched control are shown. Below the qPCR expression data the log_2 fold-change observed in the scRNA seq dataset is listed, for comparison. **(B)** Expression of IL1β mRNA in patients and controls, normalized to *HPRT*, *RPL0*, *ACTB* mRNA.

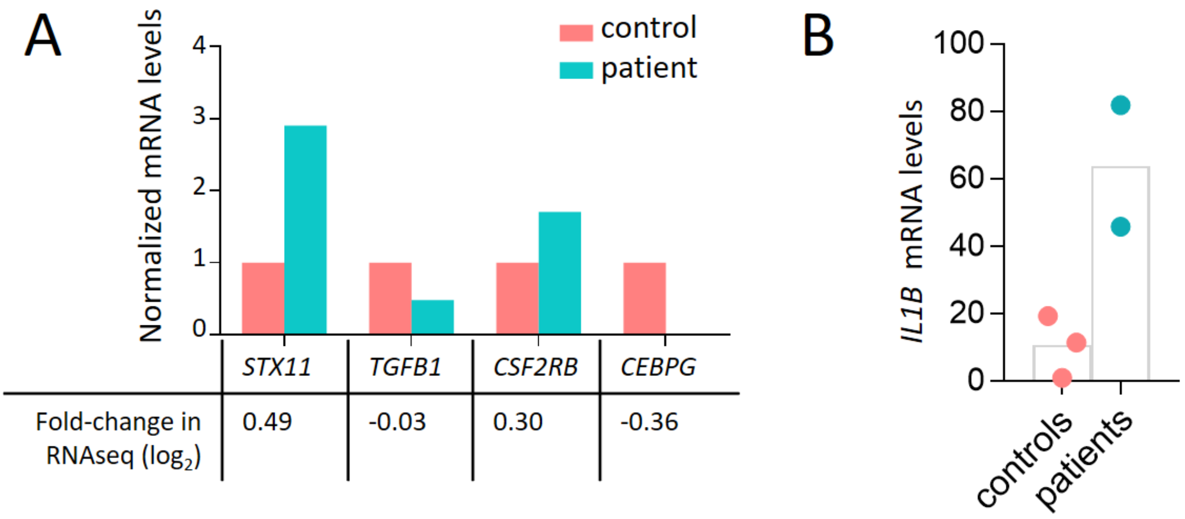

Figure S6. Differentially expressed gene sets between patient and control. Each leukocyte cluster was assessed for differentially expressed genes. Gene set analysis with GSEA was performed on the differentially expressed genes within each leukocyte cluster, with a false discovery rate of 25% used to identify up- and down-regulated pathways. **(A)** Naïve T cells, **(B)** Naïve B cells, **(C)** activated CD4 T cells, **(D)** memory B cells, **(E)** CD14^{+} monocytes, **(F)** CD1c^{+} DCs, **(G)** activated CD8 T cells, **(H)** CD56^{bright} NK cells, **(I)** CD56^{dim} NK cells, **(J)** γδ T cells, **(K)** CD16^{+} monocytes), **(L)** activated monocytes, **(M)** proliferating cells.

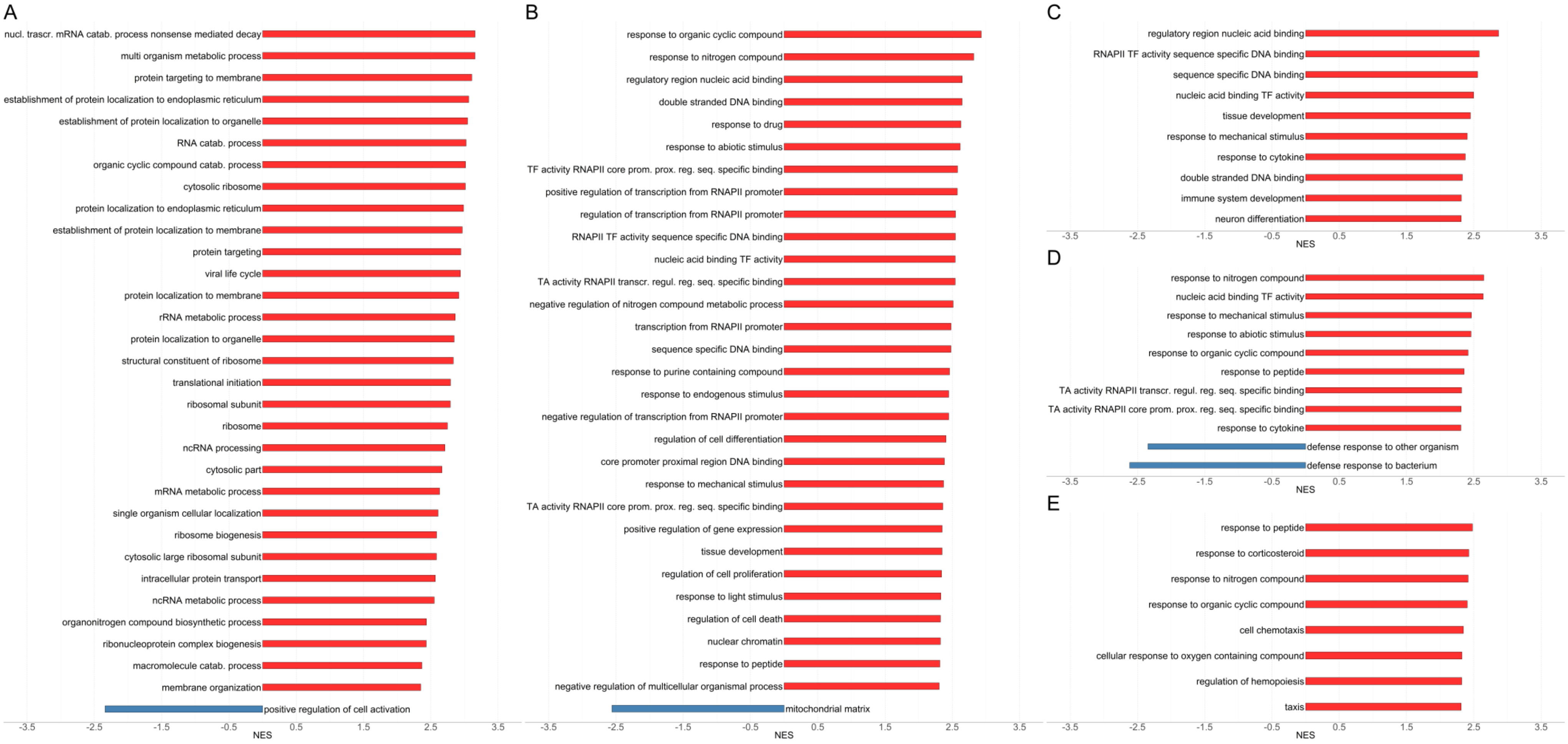

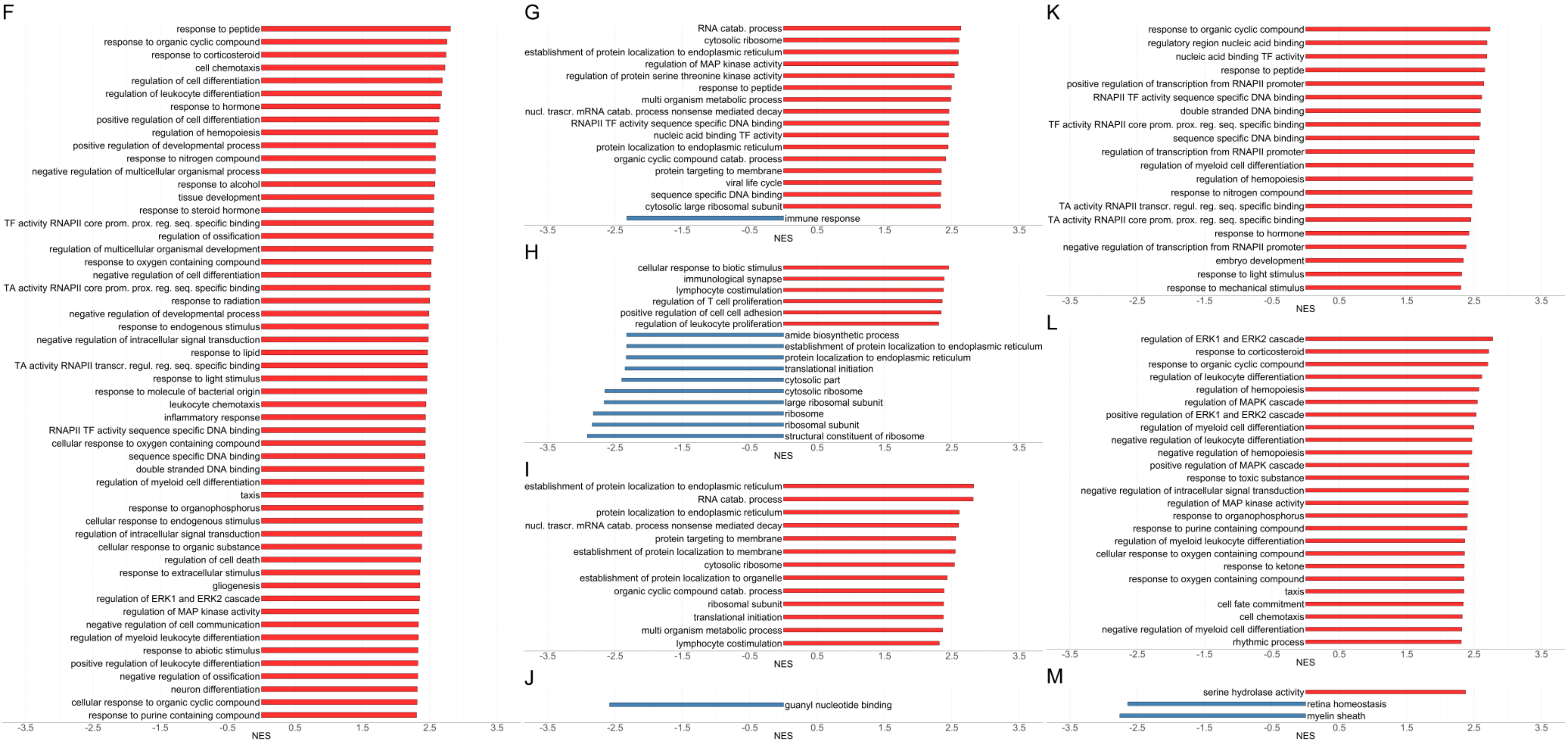
F
response to peptide
response to organic cyclic compound
response to corticosteroid
cell chemotaxis
regulation of cell differentiation
regulation of leukocyte differentiation
response to hormone
positive regulation of cell differentiation
regulation of hemopoiesis
positive regulation of developmental process
response to nitrogen compound
negative regulation of multicellular organismal process
response to alcohol
tissue development
response to steroid hormone
TF activity RNAPII core prom. prox. reg. seq. specific binding
regulation of ossification
regulation of multicellular organismal development
response to oxygen containing compound
negative regulation of cell differentiation
TA activity RNAPII core prom. prox. reg. seq. specific binding
response to radiation
negative regulation of developmental process
response to endogenous stimulus
negative regulation of intracellular signal transduction
response to lipid
TA activity RNAPII transcr. regul. reg. seq. specific binding
response to light stimulus
response to molecule of bacterial origin
leukocyte chemotaxis
inflammatory response
RNAPII TF activity sequence specific DNA binding
cellular response to oxygen containing compound
sequence specific DNA binding
double stranded DNA binding
regulation of myeloid cell differentiation
taxis
response to organophosphorus
cellular response to endogenous stimulus
regulation of intracellular signal transduction
cellular response to organic substance
regulation of cell death
response to extracellular stimulus
gliogenesis
regulation of ERK1 and ERK2 cascade
regulation of MAP kinase activity
negative regulation of cell communication
regulation of myeloid leukocyte differentiation
response to abiotic stimulus
positive regulation of leukocyte differentiation
negative regulation of ossification
neuron differentiation
cellular response to organic cyclic compound
response to purine containing compound
-3.5 -2.5 -1.5 -0.5 0.5 1.5 2.5 3.5
NES
G
RNA catab. process
cytosolic ribosome
establishment of protein localization to endoplasmic reticulum
regulation of MAP kinase activity
regulation of protein serine threonine kinase activity
response to peptide
multi organism metabolic process
nucl. trascr. mRNA catab. process nonsense mediated decay
RNAPII TF activity sequence specific DNA binding
nucleic acid binding TF activity
protein localization to endoplasmic reticulum
organic cyclic compound catab. process
protein targeting to membrane
viral life cycle
sequence specific DNA binding
cytosolic large ribosomal subunit
immune response
-3.5 -2.5 -1.5 -0.5 0.5 1.5 2.5 3.5
NES
H
cellular response to biotic stimulus
immunological synapse
lymphocyte costimulation
regulation of T cell proliferation
positive regulation of cell cell adhesion
regulation of leukocyte proliferation
amide biosynthetic process
establishment of protein localization to endoplasmic reticulum
protein localization to endoplasmic reticulum
translational initiation
cytosolic part
cytosolic ribosome
large ribosomal subunit
ribosome
ribosomal subunit
structural constituent of ribosome
-3.5 -2.5 -1.5 -0.5 0.5 1.5 2.5 3.5
NES
I
establishment of protein localization to endoplasmic reticulum
RNA catab. process
protein localization to endoplasmic reticulum
nucl. trascr. mRNA catab. process nonsense mediated decay
protein targeting to membrane
establishment of protein localization to membrane
cytosolic ribosome
establishment of protein localization to organelle
organic cyclic compound catab. process
ribosomal subunit
translational initiation
multi organism metabolic process
lymphocyte costimulation
-3.5 -2.5 -1.5 -0.5 0.5 1.5 2.5 3.5
NES
J
guanyl nucleotide binding
-3.5 -2.5 -1.5 -0.5 0.5 1.5 2.5 3.5
NES
K
response to organic cyclic compound
regulatory region nucleic acid binding
nucleic acid binding TF activity
response to peptide
positive regulation of transcription from RNAPII promoter
RNAPII TF activity sequence specific DNA binding
double stranded DNA binding
TF activity RNAPII core prom. prox. reg. seq. specific binding
sequence specific DNA binding
regulation of transcription from RNAPII promoter
regulation of myeloid cell differentiation
regulation of hemopoiesis
response to nitrogen compound
TA activity RNAPII transcr. regul. reg. seq. specific binding
TA activity RNAPII core prom. prox. reg. seq. specific binding
response to hormone
negative regulation of transcription from RNAPII promoter
embryo development
response to light stimulus
response to mechanical stimulus
-3.5 -2.5 -1.5 -0.5 0.5 1.5 2.5 3.5
NES
L
regulation of ERK1 and ERK2 cascade
response to corticosteroid
response to organic cyclic compound
regulation of leukocyte differentiation
regulation of hemopoiesis
regulation of MAPK cascade
positive regulation of ERK1 and ERK2 cascade
regulation of myeloid cell differentiation
negative regulation of leukocyte differentiation
negative regulation of hemopoiesis
positive regulation of MAPK cascade
response to toxic substance
negative regulation of intracellular signal transduction
regulation of MAP kinase activity
response to organophosphorus
response to purine containing compound
regulation of myeloid leukocyte differentiation
cellular response to oxygen containing compound
response to ketone
response to oxygen containing compound
taxis
cell fate commitment
cell chemotaxis
negative regulation of myeloid cell differentiation
rhythmic process
-3.5 -2.5 -1.5 -0.5 0.5 1.5 2.5 3.5
NES
M
serine hydrolase activity
retina homeostasis
myelin sheath
-3.5 -2.5 -1.5 -0.5 0.5 1.5 2.5 3.5
NES
